# Using a Contemporary Portable Metabolic Gas Exchange System for Assessing Energy Expenditure: A Validity and Reliability Study

**DOI:** 10.3390/s23052472

**Published:** 2023-02-23

**Authors:** Holly L. McClung, William J. Tharion, Leila A. Walker, Maxwell N. Rome, Reed W. Hoyt, David P. Looney

**Affiliations:** 1 Military Performance Division, US Army Research Institute of Environmental Medicine (USARIEM), 10 General Green Ave., Natick, MA 01760, USA; 2 Oak Ridge Institute for Science and Education (ORISE), 1299 Bethel Valley Rd., Oak Ridge, TN 37830, USA; 3 Military Nutrition Division, US Army Research Institute of Environmental Medicine (USARIEM), 10 General Green Ave., Natick, MA 01760, USA

**Keywords:** metabolic training sensor, indirect calorimetry, energy expenditure, oxygen uptake

## Abstract

There are several methods available to assess energy expenditure, all associated with inherent pros and cons that must be adequately considered for use in specific environments and populations. A requirement of all methods is that they must be valid and reliable in their capability to accurately measure oxygen consumption (VO_2_) and carbon dioxide production (VCO_2_). The purpose of this study was to evaluate the reliability and validity of the mobile CO_2_/O_2_ Breath and Respiration Analyzer (COBRA) relative to a criterion system (Parvomedics TrueOne 2400^®^, PARVO) with additional measurements to compare the COBRA to a portable system (Vyaire Medical, Oxycon Mobile^®^, OXY). Fourteen volunteers with a mean of 24 years old, body weight of 76 kg, and a VO_2peak_ of 3.8 L∙min^−1^ performed four repeated trials of progressive exercises. Simultaneous steady-state measurements of VO_2_, VCO_2_, and minute ventilation (V_E_) by the COBRA/PARVO and OXY systems were conducted at rest, while walking (23–36% VO_2peak_), jogging (49–67% VO_2peak_), and running (60–76% VO_2peak_). Data collection was randomized by the order of system tested (COBRA/PARVO and OXY) and was standardized to maintain work intensity (rest to run) progression across study trials and days (two trials/day over two days). Systematic bias was examined to assess the accuracy of the COBRA to PARVO and OXY to PARVO across work intensities. Intra- and inter-unit variability were assessed with interclass correlation coefficients (ICC) and a 95% limit of agreement intervals. The COBRA and PARVO produced similar measures for VO_2_ (Bias ± SD, 0.01 ± 0.13 L·min^−1^; 95% LoA, (−0.24, 0.27 L·min^−1^); R^2^ = 0.982), VCO_2_ (0.06 ± 0.13 L·min^−1^; (−0.19, 0.31 L·min^−1^); R^2^ = 0.982), V_E_ (2.07 ± 2.76 L·min^−1^; (−3.35, 7.49 L·min^−1^); R^2^ = 0.991) across work intensities. There was a linear bias across both the COBRA and OXY with increased work intensity. The coefficient of variation for the COBRA ranged from 7 to 9% across measures for VO_2_, VCO_2_, and V_E_. COBRA was reliable across measurements for VO_2_ (ICC = 0.825; 0.951), VCO_2_ (ICC = 0.785; 0.876), and V_E_ (ICC = 0.857; 0.945) for intra-unit reliability, respectively. The COBRA is an accurate and reliable mobile system for measuring gas exchange at rest and across a range of work intensities.

## 1. Introduction

Several methods exist to assess accurate energy expenditure, from direct measurements using whole-room calorimeters for the doubly labeled water methodology to assess the multiple day free-living energy expenditure, to less cumbersome and less expensive indirect methods using stationary metabolic carts, mobile body-worn sensors, or heart rate monitoring systems. All methods are associated with inherent limitations (e.g., cost, limited activity assessment, daily urine samples, etc.) that must be considered and matched to desired outcomes.

Providing modern, mobile solutions to accurately assess energy expenditure in settings beyond the laboratory could enable widespread use and may bring a contemporary, individualized understanding of energy utilization, weight maintenance monitoring, and enable personalized training optimization. New mobile sensor systems are entering the market as applications to smart phones or as body-worn metabolic monitoring systems. Many of these systems or devices are easy to use and offer the ability to sample energy expenditure under free-living conditions for ‘real-time’ monitoring. A persistent issue with mobile monitoring sensors has been the lack of precision and accuracy [1,2,3,4,5]. This limitation as well as cost has traditionally prohibited the widespread application of mobile metabolic system use as training tools for athletes, the military, and individuals striving to manage individualized performance and body weight goals. Ideally, such monitors could aid individuals whose goal is to enhance performance through optimized training specific to sports with varying intensities. For example, they could be used in endurance activities (over multiple hours), such as ultra-endurance events like ultramarathons or multi-stage bicycling races (e.g., the Tour de France), and elite multi-day military training exercises where the exercise intensity levels vary over the training event [6,7,8,9]. Knowledge of substrate utilization could enhance training effectiveness and event performance. Sensors with minimal burden used during habitual training could provide an advantage. However, there is a need to replace the use of heart rate monitors that aid in determining exercise/work intensity but fall short of providing accurate assessments of energy expenditure [10].

Researchers traditionally use indirect calorimetry as an accurate method to measure metabolic rate at rest and during exercise through quantitative measurements of O_2_ consumption (VO_2_) and CO_2_ production (VCO_2_). This methodology can be used to determine individualized exercise intensity (%VO_2max_) and, indirectly, substrate utilization (carbohydrate and fat) [11,12,13] for use in training optimization. Conventional metabolic assessments occur under controlled laboratory environments with stationary metabolic carts or through use of cumbersome body-worn metabolic monitoring systems [4,11]. Adopting any new method of energy assessment requires confidence in the system of choice through analysis of the systems’ validity and reliability, reported as variance in relation to a criterion or gold standard method. Comparisons between methods (or systems) focus on coefficients of variation (CV) for maximal values of VO_2_, VCO_2,_ and minute ventilation (V_E_), as well as within-subject variability. Metabolic cart systems have reported CV values of 4–14% for VO_2_, 5.5% for VCO_2_, 8.5% for V_E_, and 5.2% for within-subject (intra-individual) variability [11,14]. The reliability of automated metabolic cart systems has appeared to be greatly influenced by biological (inter-individual) variability even under the strictest of laboratory-controlled conditions. Reports suggest ≤10% of the total error in measurement of VO_2_ to be associated with technical issues of measurements innate to the systems [14]. However, tighter VO_2_ measurements (coefficients of variation as low as <3%) have been reported with repeated measurements during steady-state, sub-maximal exercise [14]. Management of intra-individual variability includes standardizing sampling intervals (15–20 s average or 5–8 breaths) to allow for more precise data comparison across ‘real-life’ laboratory conditions [11].

Recently a novel, low-cost, body-worn metabolic gas measurement system, the CO_2_/O_2_ Breath and Respiration Analyzer (COBRA), was developed and has been described from an engineering perspective [15,16,17]. The initial work by Candell et al., [16] describes the unique design of this mobile COBRA system’s passive proportional side stream sampling innovation, complete with a detailed description of the design specifications and in-depth description of the sensor components [17]. To date, limited validation testing has been assessed for the COBRA, and to our knowledge, no physiological assessment of the COBRA validity and reliability for indirect calorimetry use in humans has been completed.

The purpose of the current study was to determine the accuracy and reliability of the COBRA against a criterion stationary system (ParvoMedics TrueOne 2400) using a test–retest design. Additionally, this design allowed for a comparison of the COBRA to a commercially available and widely used mobile system (Oxycon Mobile^®^) under similar conditions to assess the viability of the COBRA as a potentially cost-effective, easy-to-use replacement system.

## 2. Materials and Methods

### 2.1. Study Design 

**Figure 1:**
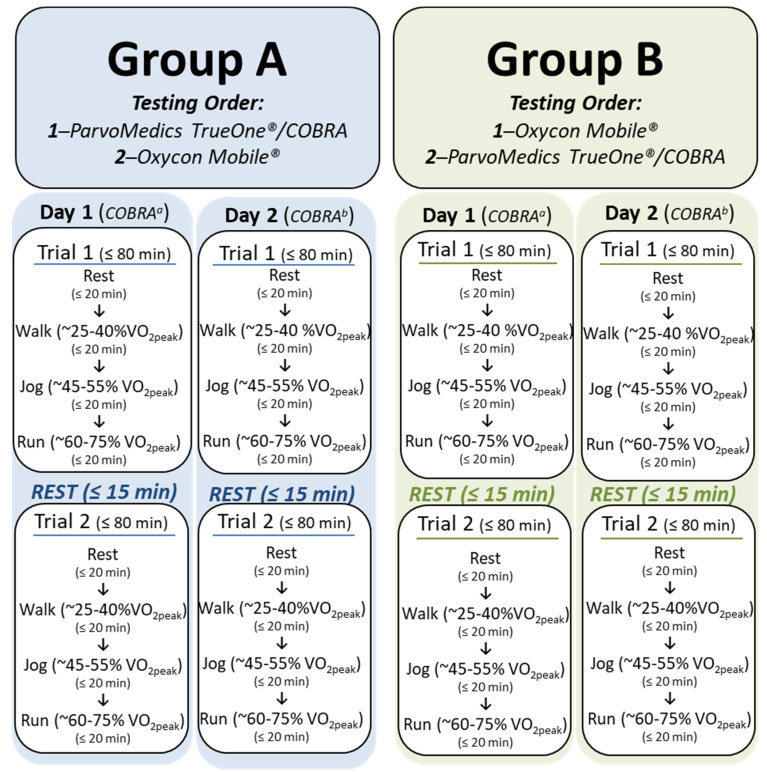
ParvoMedics TrueOne^®^ was used as the criterion device and tested at the same time as the COBRA system to allow for simultaneous breath sampling. Each rest and exercise bout was ≤20 min to include: ~5 min to reach a steady state; 7 min of data collection with system #1; 7 min of data collection with system #2 (daily testing ≤ 3½ h total; ≤2 h of exercise). COBRA*^a^* ^or *b*^ denote the alternation of sensor units used in data collection; VO_2peak_ signifies peak oxygen uptake. The Oxycon Mobile^®^ system was tested immediately before or after the COBRA system as the design of the facemask would not allow for this system to be tested simultaneously with the COBRA system.

The randomized cross-over study was conducted over three days: the baseline and two non-consecutive study days. Baseline assessments, consisting of anthropometric data collection to characterize the study participants, the determination of VO_2peak_, and equipment familiarization, were followed by two days of data collection in a temperature-controlled laboratory (20 ± 0.5 °C; 45 ± 22 % RH). Four testing trials were conducted over the two days (two trials/test day) alternating between two metabolic system configurations (Figure 1). To control for possible order effects, participants were randomly assigned to either Group A (device test order: PARVO/COBRA, OXY) or Group B (device test order: OXY, PARVO/COBRA) at baseline (Figure 1). This testing trial design allowed for the assessment of the COBRA system validity and intra-unit variability (i.e., reproducibility of the same measurement using the same device under similar conditions) and inter-unit variation of COBRA system units over trials.

Baseline characterization measurements were collected in shorts, t-shirts, and socks and included height using a portable stadiometer (model 217, SECA, Chino, CA, USA), body mass using a calibrated electronic scale (model DS6150, Doran, Batavia, IL, USA), and body composition using dual energy X-ray absorptiometry (DEXA, DPX-IQ, Lunar Corporation, Madison, WI, USA). Fitness was assessed through VO_2peak_ using indirect calorimetry measured with the PARVO metabolic cart.

Peak oxygen consumption was assessed using a continuous incremental uphill treadmill running protocol [18], which began after a 5 min warm-up period. Briefly, participants walked on the treadmill at 0% grade for 2 min at 1.3 m·s^−1^, then for 3 min at the running speed of 2.2–2.9 m·s^−1^ (based on self-reported 2-m run time). Following the warm-up, the speed of the treadmill remained the same, but the grade was increased to 5% for 2 min and then increased by 2.5% grade every 2 min thereafter until voluntary exhaustion. The VO_2peak_ was defined as the point when the participant’s oxygen consumption increased by less than 2 mL·kg^−1^·min^−1^ following an increase in workload, the heart rate achieved was more than the age-predicted maximum, and/or the respiratory exchange ratio exceeded 1.0 [18].

**Figure 2:**
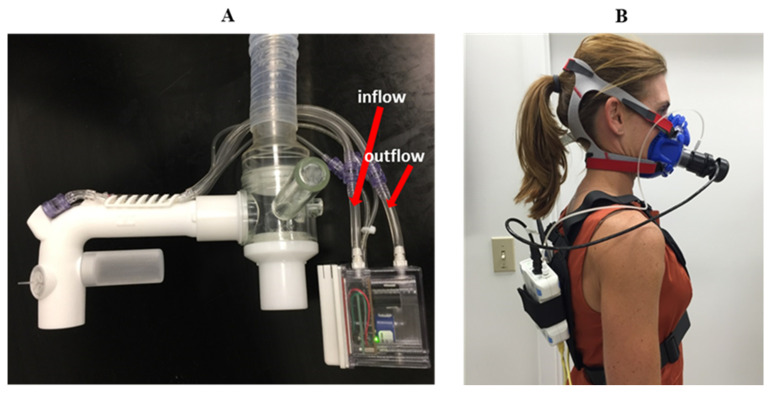
Sensor system configurations: (**A**) COBRA system attached to ParvoMedics TrueOne^®^ valve and hose, which leads to the metabolic cart (not shown); (**B**) Oxycon Mobile^®^ sensor and data units (e.g., SBx and DEX, respectively) harnessed with the turbine attachment to the face mask.

The VO_2peak_ values were used to determine standardized work rates during test trials (walk: 20–40% VO_2peak_; jog: 45–55% VO_2peak_; run 60–75% VO_2peak_) based upon the American College of Sports Medicine Guidelines for aerobic training [18]. Participants were familiarized with the calculated work rates (~5 min at each work rate stage) to finish baseline testing. Finally, during the baseline condition, participants were familiarized and fit for use with the two metabolic system configurations PARVO/COBRA and OXY (Figure 2). 

Participants were instructed to refrain from exercise, caffeine, and nicotine intake for at least 24 h prior to testing. Participants reported to the laboratory for all test days fasted (>10 h) at a consistent time between 6:30 and 8:00 a.m. and were instructed to wear standard physical training attire. Body weight was assessed prior to data collection for use in unit expression of metabolic variables.

### 2.2. Participants

Fourteen healthy volunteers (13 men, 1 woman; 24 ± 6 y (mean ± SD), 76 ± 13 kg BW, 24 ± 5% total body fat, 55 ± 9 kg lean body mass, VO_2peak_ 3.8 ± 0.7 L∙min^−1^) were recruited to participate in data collection. Attempts to recruit both men and women were made, but only one woman completed the study (one started and later withdrew). Differences in body size and total lung volume were considered the predominant determinants of the COBRA’s performance. Hence, we sought a diverse set of volunteers with respect to body size and total lung volume to ensure the COBRA system would perform over a range of body sizes irrespective of sex. Participants were recreationally active civilian and military individuals who engaged in aerobic activity 3–4 d/wk.

Potential study participants were formally briefed on the protocol requirements and provided written informed consent prior to data collection. This study was reviewed and approved by the US Army Medical Research and Development Command’s Institutional Review Board (IRB); all data collection occurred at the US Army Research Institute of Environmental Medicine (USARIEM; Natick, MA, USA) between December 2017 and August 2018. Individuals were not eligible for study participation if they were unable to exercise, were pregnant, had difficulty breathing through a mouthpiece, or reported having issues with claustrophobia. Investigators adhered to the policies regarding the protection of human subjects as prescribed in Army Regulation 70–25, and the research was conducted in adherence with the provisions of 32 CFR Part 219.

### 2.3. Sensor Systems

Determination of the COBRA validity of measurements was accomplished by testing the COBRA to the ParvoMedics TrueOne 2400 (Parvo Medics; Salt Lake, UT, USA; PARVO) simultaneously. To demonstrate the potential use of the COBRA in place of a widely used, relatively reliable, and valid commercial off-the-shelf (COTS) system, the metabolic measures from Oxycon Mobile^®^ (Vyaire Medical, Inc., Mettawa, IL, USA; OXY) were compared to PARVO. The comparison of measures obtained from the OXY to the PARVO/COBRA was completed in sequence, as the three systems could not be worn at the same time.

The PARVO metabolic cart uses a mixing chamber design, rather than a breath-by-breath approach and has been consistently demonstrated to be valid and reliable under rest and exercise conditions [11,19,20,21,22]. Results of an inter-unit variability study conducted across two PARVO systems documented total submaximal within-subject variations for VO_2_, VCO_2_, and V_E_ of ~4% with inter-unit technological errors of ~1.5% [18]. Such findings are comparable to the criterion Douglas bag method with the added benefit of automation [18]. The PARVO was used in this testing assessment as the criterion measure.

**Figure 3:**
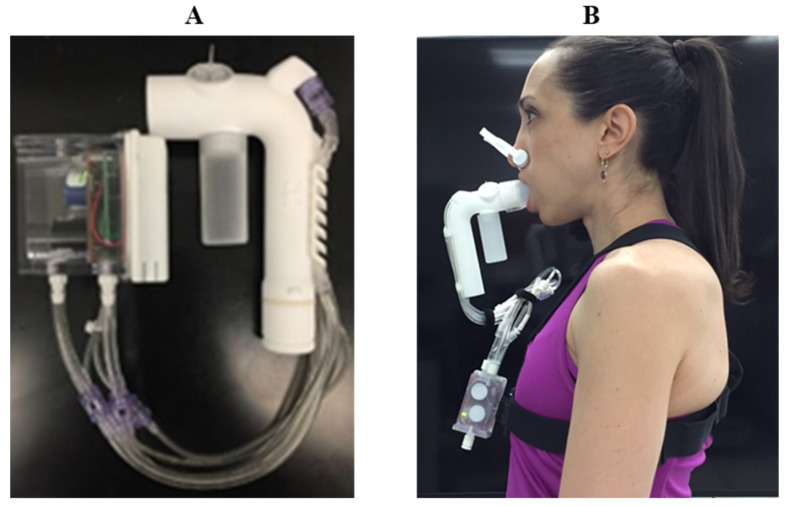
(**A**) CO_2_/O_2_ Breath and Respiration Analyzer, COBRA with (**B**) the chest mount harness in use.

The COBRA (Figure 3; patent US 10,638,956B2, Systems, apparatus, and methods related to modeling, monitoring, and/or managing metabolism, May 2020) [15,16,17] is a portable, wearable mixing chamber system designed to measure gas exchange in free-living individuals described in detail by Candell, et al. [16,17] with system specifications described in the patent document [15]. The two COBRA prototype units used in testing were manufactured through 3D printing and hand assembly at the Massachusetts Institute of Technology Lincoln Laboratory (MITLL). Briefly, the COBRA is comprised of two components: a small mixing chamber (complete with O_2_, CO_2_, temperature and flow sensors, a microprocessor, and battery) and a J-shaped flowtube connected to the mixing chamber by small, flexible plastic tubing (Figure 3). The flowtube is fitted with a rubber mouthpiece. COBRA data can either be viewed live through a USB connection or Bluetooth-connected device. Data can also be stored onboard in non-volatile memory for computer download using COBRA-specific software.

Measurements between the criterion method (PARVO) and COBRA were made in series (Figure 2). The effects of the added dead space associated with routing expired breath through the COBRA flowtube prior to entering the PARVO system were accounted for in the final calculations. Calibration of the flow was completed with the PARVO and COBRA in series using a 3 L calibration syringe. Gas calibration was performed separately for each device before and after each testing trial. The series measurement between sensors was selected to minimize biological day-to-day variation.

The OXY is a COTS system that is portable and wireless, allowing for breath-by-breath gas exchange measurement both inside and outside the laboratory. The OXY acquires data utilizing an ‘open system’ that may be viewed through live streaming (e.g., within range of the receiver) or saved to an onboard data file. The OXY has been shown to provide reasonably valid and reliable data across a range of metabolic work intensities. Validity and reliability comparisons between the OXY system and the Douglas bag method found the OXY in general to demonstrate accuracy over a wide range of VO_2_, while VCO_2_ was overestimated (3–7%) [4]. The V_E_ measurements were accurate at submaximal work rates; however, underestimations (4–8%) occur with increased work intensity [4].

For use in testing, the OXY system was calibrated daily prior to testing and according to the manufacturer’s specified practices. Measurements with the OXY were made successively, either prior to or following the PARVO/COBRA measurements (Figure 1). For continuity, participants used the same OXY system for each testing trial and day.

### 2.4. Protocol Assessment

COBRA sensor agreement was assessed for three primary measurements: VO_2_, VCO_2_, and V_E_ as compared to the criterion measure (PARVO) and OXY. To maintain consistency for reliability measures, data collection routines were repeated for the two test groups (A and B) over the two non-consecutive test days with participants completing a total of four bouts of incremental exercise over the entire study (Figure 1). Each test day consisted of two bouts of exercise (Trial 1 and Trial 2) separated by a 10–20 min of seated rest. Participants remained fasted until the end of testing Trial 2 each day (≤3½ h). Water was provided ad libitum throughout the testing sessions.

The testing routine started on Test Day 1, when participants arrived at the lab fasted and were fitted to a metabolic analyzer system (A or B; Figure 2) by study staff. Participants sat quietly in an upright position for ~10 to 15 min to bring them to a resting state. Trial 1 data collection was initiated once a resting state was achieved (metabolic equivalent, MET ≤ 1.0 [23]; resting heart rate, HR_Rest_), and the rest period concluded once data collection was completed with both metabolic sensor system configurations (i.e., PARVO/COBRA and OXY). Participants were then moved to the treadmill where they performed light-to-moderate work: walk (~25–40% VO_2peak_) → jog (~45–55% VO_2peak_) → run (~60–75% VO_2peak_). Work intensity levels were calculated based on peak oxygen determinations at baseline. Incremental progression of work was the safest method to prevent participant injury, as the progressive work intensity did not require recovery from a higher workload to assess respiratory measurements of lower workloads.

Participants performed at each prescribed work level for a total of ~20 min, starting with a warm-up to reach a steady-state status (~5 min). Data collection with system #1 was then collected (~5 to 7 min), followed by data collection with system #2 (~5 to 7 min), before progressing to the next level of increased work (Figure 1). Response to prescribed aerobic activity was monitored through the live streaming of the data from the metabolic sensor systems and the use of a commercial heart rate monitoring system (Polar T31^®^ Heart Rate Sensor; Kempele, Finland). Data were averaged over the 3-to-4-min steady-state periods at rest and for each work intensity.

Once participants completed the three phases of exercise, they were given a 10 to 15 min rest period (i.e., sit, bathroom use, hydration, etc.) before the initiation of Trial 2. Trial 2 directly replicated Trial 1 to include the same incremental increase in work intensity (i.e., rest →walk → jog → run) and identical timing (Figure 1). Participants completing Trial 2 completed testing for Test Day 1.

Follow on testing continued with participants returning on a non-consecutive day (e.g., Test Day 1 = Thursday; Test Day 2 = Tuesday) to complete Test Day 2 data collection. Participants replicated physical activities, work intensities (e.g., speed/grade), and timing from Test Day 1 (i.e., two incremental test trials with a 10–15 min rest in between) with the only variation from Test Day 1 being the use of a different COBRA unit (a or b) to collect data (Figure 1).

### 2.5. Statistical Analyses

The sample size was calculated using SPSS (Sample Power; SPSS; Chicago, IL, USA), taking into consideration accuracy and an acceptable level of agreement across three primary variables: VO_2_, VCO_2_, and V_E_. Statistical analyses were conducted using R (Version 3.3.1; R Foundation for Statistical Computing; Vienna, Austria [24]. Data are presented as mean ± SD unless otherwise noted. Bland–Altman-like plots [25] were generated to illustrate the differences from the criterion measure (PARVO) for the COBRA and OXY when measuring VO_2_, VCO_2_, and V_E_. Bias and 95% limits of agreement (95% LoA) were calculated along with the marginal coefficient of determination (R^2^) using a multi-level model approach to account for the hierarchical structure of the dataset [26]. Random effects of participant within activity on intercepts for the analyses of agreement were included. Coefficient of variation and intraclass correlation coefficients (ICC) were used to evaluate the reliability of the COBRA systems across study trials (intra-unit) and across days (inter-unit) across different variables. Within-day, within-activity random effects and within-trial, within-activity random effects of each participant were included for these analyses, respectively. The 95% confidence interval for each ICC was determined using the bootstrap percentile method [27].

## 3. Results

For validation comparison, Table 1 delineates mean values of physiological response measurements for each sensor system.

**Figure 4:**
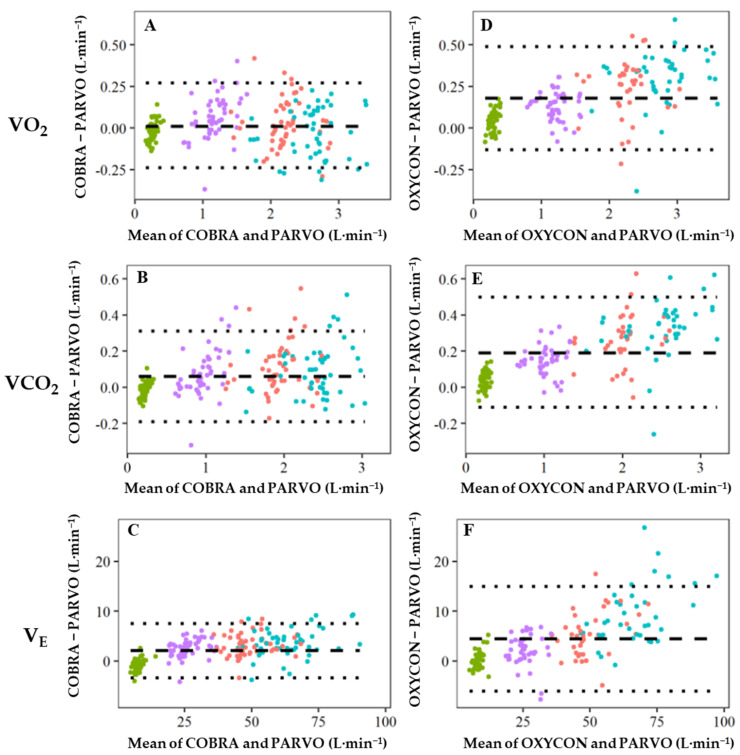
Bland–Altman-like plot of agreement between (**A**,**D**) O_2_ consumption (VO_2_), (**B**,**E**) CO_2_ production (VCO_2_), and (**C**,**F**) minute ventilation (V_E_) measured by (**A**–**C**) CO_2_/O_2_ Breath and Respiration Analyzer (COBRA) and (**D**–**F**) Oxycon Mobile^®^ (OXY) as compared to the ParvoMedics TrueOne 2400 (PARVO). Means ± SD across incremental exercise: rest (green), walk (purple), jog (red), and run (teal); dashed line, bias; dotted lines, 95% limits of agreement.

Bland–Altman-like plots (Figure 4) demonstrate the agreement across the three primary measurements: VO_2_, VCO_2_, and V_E_ between the COBRA and PARVO (panels A–C) and the OXY and PARVO (panels D–F) with variable work intensities.

Table 2 shows overall measurement bias and agreement for the COBRA and OXY as compared to the PARVO across the primary measures. For the COBRA and OXY, VO_2_, VCO_2_, and V_E_ were in close agreement with the criterion measure (PARVO) across work intensities, and the error was uniform over the range examined. The OXY measurements for VO_2_, VCO_2_, and V_E_ were similar. For both COBRA and OXY, the measurement errors expanded as exercise intensity increased (Figure 4). COBRA was in high agreement (R^2^= 0.982–0.991) with the PARVO across each activity of VO_2_, VCO_2_, and V_E_.

The COBRA showed good within- and between-system reliability (VO_2_ ICC were 0.825 and 0.951, VCO_2_ ICC were 0.785 and 0.876, respectively). The same was true for V_E_ (ICC = 0.857; 0.945) for inter- and intra-unit reliability, respectively (Table 3).

Use of the COBRA resulted in an overall CV (inter- and intra-unit) in the range of 7–9% across measures (Table 4) and within-unit variability was 4.3 % for VO_2_, 7.0% for VCO_2_, and 4.7% for V_E_.

## 4. Discussion

Mobile metabolic monitoring systems that can accurately assess energy expenditure have the potential to expand health monitoring and optimize training and energy needs. The major finding of this study was a newly developed portable metabolic system, the COBRA, provides acceptable measurements for gas exchange at rest and across work intensities. This feature is important as previously noted for the purpose of providing individualized information regarding adaptions to training [28]. From Table 1 it may be observed that the COBRA values for the main measures of interest—VO_2_, VCO_2_, and V_E_—were not practically different from the PARVO criterion measure, and were closer to the PARVO in values across all exercise activities than those obtained from the OXY, the comparison portable device. However, it has to be acknowledged that part of the difference in the OXY values is likely due to the methodological limitation of not being able to assess the PARVO and OXY simultaneously. Nevertheless, strict control in replicating the trials with sufficient recovery between Trial 1 and Trial 2 on a particular day was adhered to, and counterbalancing to control for order effects was employed. Therefore, since the COBRA values consistently matched the PARVO values more closely than the OXY values did, this provides a first-glance justification of use of the COBRA as a replacement system for the OXY as a field, non-tethered assessment device to obtain these respiratory measurements. Examination of reliability metabolic measurements from Table 3 confirms the intra-test reliability was high for VO_2_ and V_E_ with ICC values of approximately 0.95 for these two measures. The VCO_2_ was lower at approximately 0.88 but is still acceptable for the general use of a fitness tracking assessment tool. The inter-test reliability (i.e., the assessment of measurements between devices) was lower as might be expected of 3D-printed, hand-assembled prototypes where slight differences in the construction and assembly are possible. Use of injected molded or other manufacturing techniques where a series of product specifications are set and controlled for through a rigorous inspection control process would likely bring the reliability of measures between devices closer to that observed of the test–retest design observed here within the system devices.

With regard to the validity measurements, the COBRA was in close agreement with the well-accepted PARVO stationary metabolic system across rest and three incremental work intensities as measured by VO_2_, VCO_2_, and V_E_. As noted in Table 2, all measures of variation are small when comparing the COBRA to the PARVO and are superior to measures of the OXY with the PARVO. The caveat regarding the methodological limitation described needs to be considered when comparing validity measures of COBRA to OXY. Regardless, even examining the R^2^ measures of just the COBRA to the PARVO shows impressive replication of results, since for VO_2_, VCO_2_, and V_E_, the R^2^ values were all over 0.98. These findings provide useful laboratory evidence to support research-grade use of the COBRA systems under controlled settings.

The Bland–Altman-like plots (Figure 4A–C) illustrate the differences between the COBRA and the criterion measure (PARVO) with an acceptable accuracy across the four activity intensities. That is, the error values were small and there was no systematic error. Evidence of systematic error would be seen if there was a pattern of dots in Figure 4A–C rising or falling based on the type of exercise (the varying colored dots). In fact, there was a pattern seen with the OXY (Figure 4D–F), specifically with the run (i.e., teal dots), which showed a rise over the other exercise conditions.

The performance of the COBRA system compares favorably to other stationary and portable metabolic systems under controlled conditions: with lower intensity work, it showed a 3 to 7% difference [11,29,30] and a 5–9% difference at high to maximal intensity [4,28]. The COBRA system was the most accurate for metabolic measurements taken at rest, likely due to the more uniform and stable breathing by participants as well as the mathematical reality of smaller values. The intra- and inter-unit reliabilities observed are comparable to other stationary and mobile systems [4,30]. The importance of a reliability and validity study prior to use of a new piece of equipment, whether for research or operational use, cannot be underestimated. The design of this study incorporated activities over a range of metabolic demand activities, and the results included herein meet these basic objectives.

### 4.1. Practical Applications

Findings from this work validate the use of the COBRA sensor system in place of the OXY or similar COTS metabolic sensor systems to provide a ‘real-time’ energy expenditure assessment outside of the confines of the laboratory. Potential commercialization of the COBRA may provide a low-cost, easy-to-use tool for the lay population to use to aid recreationally active (ultra-endurance) athletes in individualized training optimization through adjustments in diet to meet the nutrition demands during training and recovery periods. Likewise, the COBRA may have an operational use by the military to assist soldier trainings toward peak performance and mission readiness through optimizing training, nutrition, and rest. Furthermore, the use of the COBRA is easier to use than a system like the OXY, where a mask must be properly fitted over the head with no leaks around the edges of the mask. Use of the COBRA, in contrast, requires the user to simply put the mouthpiece in their mouth and a nose-clip over their nose to ensure no air escapes from the nose.

### 4.2. Limitations

Although significant efforts were made to design a well-controlled study, limitations remain. The innate differences between the breath-by-breath (OXY) and mixing chamber systems (PARVO and COBRA) did not allow for the direct comparison of COBRA with the OXY. The breath-by-breath design in these devices creates a time differential between flow and gas measurements, which require a temporal matching of volume (flow) and expired gas fractions to calculate VO_2_ and VCO_2_. The COBRA uses a small mixing chamber and longer sampling times are needed to ensure the full flush of the mixing chamber. Due to system engineering (e.g., mixing chamber systems vs. breath-by-breath system), the systems were not in series; therefore, the OXY system was tested a few minutes before or after the series sampling of PARVO/COBRA systems for each type of work intensity depending on randomization. While the two COBRA prototypes were built to the specifications listed in the patent, systematic testing across multiple devices is still required. Future efforts should look to expand validation across environmental extremes (e.g., temperature, humidity, and pressure) to better understand the environmental range in which the COBRA function is most acceptable. While this testing has proven to be valid on a single or multiple COBRAs, a study with low-rate initial production using the exact manufacturing methods should be conducted to ensure reliability and validity across test devices to ensure that the process of manufacturing does not introduce its own set of errors in the production of this intricate device. Finally, an assumption was made that sex should not make a difference in the performance of the COBRA system once body size and lung volume were accounted for. This assumption should be verified in a study powered to assess potential sex differences.

## 5. Conclusions

Metabolic testing is moving beyond the confinements of the laboratory. The results of this study indicate that the COBRA is an accurate and precise instrument for determining energy expenditure, and its performance is equal to or better than a COTS system, with the estimated price point of the COBRA system to be below current COTS systems to allow for use by a greater range of individuals.

## Figures and Tables

**Table 1:** Physiological responses measured during rest and three work intensities across the metabolic sensor system.

Measurement ^1^	PARVO	COBRA	OXY
**VO_2_ (STPD, L** **·** **min^−1^)**			
Rest	0.27 ± 0.05	0.26 ± 0.07	0.32 ± 0.08
Walk	1.14 ± 0.20	1.20 ± 0.26	1.27 ± 0.19
Jog	2.19 ± 0.33	2.22 ± 0.32	2.40 ± 0.35
Run	2.62 ± 0.40	2.60 ± 0.42	2.94 ± 0.46
**VCO_2_ (STPD, L** **·** **min^−1^** **)**			
Rest	0.22 ± 0.04	0.21 ± 0.06	0.26 ± 0.05
Walk	0.93 ± 0.17	1.01 ± 0.23	1.08 ± 0.18
Jog	1.89 ± 0.27	1.99 ± 0.30	2.13 ± 0.31
Run	2.34 ± 0.36	2.43 ± 0.39	2.68 ± 0.42
**V_E_ (BTPS, L** **·** **min^−1^** **)**			
Rest	8.16 ± 1.36	7.28 ± 1.93	10.42 ± 2.25
Walk	24.93 ± 4.49	27.51 ± 5.13	33.44 ± 5.56
Jog	47.57 ± 7.62	50.75 ± 7.91	64.73 ± 11.87
Run	61.60 ± 10.30	65.25 ± 11.23	86.05 ± 15.70
**F_E_O_2_**			
Rest	0.1769 ± 0.0043	0.1751 ± 0.0058	0.1711 ± 0.0047
Walk	0.1652 ± 0.0049	0.1668 ± 0.0054	0.1627 ± 0.0045
Jog	0.1645 ± 0.0043	0.1659 ± 0.0046	0.1637 ± 0.0056
Run	0.1675 ± 0.0039	0.1693 ± 0.0042	0.1669 ± 0.0045
**F_E_CO_2_**			
Rest	0.0278 ± 0.0037	0.0291 ± 0.0048	0.0299 ± 0.0043
Walk	0.0380 ± 0.0043	0.0373 ± 0.0052	0.0400 ± 0.0042
Jog	0.0404 ± 0.0042	0.0403 ± 0.0048	0.0400 ± 0.0045
Run	0.0386 ± 0.0040	0.0383 ± 0.0047	0.0366 ± 0.0040
**RER**			
Rest	0.82 ± 0.10	0.81 ± 0.10	0.83 ± 0.07
Walk	0.82 ± 0.04	0.84 ± 0.06	0.85 ± 0.06
Jog	0.87 ± 0.04	0.90 ± 0.05	0.89 ± 0.05
Run	0.89 ± 0.04	0.94 ± 0.05	0.91 ± 0.05
**Rf (breaths·** **min^−1^** **)**			
Rest	17 ± 4	19 ± 5	18 ± 4
Walk	28 ± 6	31 ± 7	27 ± 6
Jog	36 ± 7	40 ± 7	38 ± 6
Run	42 ± 8	46 ± 8	47 ± 8

^1^ Values are means ± SD. PARVO, ParvoMedics cart system; COBRA, CO_2_/O_2_ Breath and Respiration Analyzer; OXY, Oxycon Mobile^®^ portable system; BTPS, body temperature pressure saturated; STPD, standard temperature pressure dry; VO_2_, oxygen uptake; VCO_2_, carbon dioxide production; V_E_, minute ventilation; F_E_O_2_, fraction of oxygen in expired air; F_E_CO_2_, fraction of carbon dioxide in expired air; RER, respiratory exchange ratio; Rf, respiratory rate.

**Table 2:** Overall agreement and error across various work intensities of CO_2_/O_2_ Breath and Respiration Analyzer (COBRA) and Oxycon^®^ (OXY) portable system to ParvoMedics (PARVO) cart system.

Measurement	PARVO vs. COBRA	PARVO vs. OXY
**VO_2_ (L·min^−1^)**		
Bias ± SD	0.01 ± 0.13	0.18 ± 0.16
95% LoA	−0.24, 0.27	−0.13, 0.49
R^2^	0.982	0.985
**VCO_2_ (L·min^−1^)**		
Bias ± SD	0.06 ± 0.13	0.19 ± 0.16
95% LoA	−0.19, 0.31	−0.11, 0.50
R^2^	0.982	0.987
**V_E_ (L·min^−1^)**		
Bias ± SD	2.07 ± 2.76	4.45 ± 5.37
95% LoA	−3.35, 7.49	−6.07, 14.98
R^2^	0.991	0.974

**Table 3:** Intraclass correlation coefficients with 95% confidence intervals for CO_2_/O_2_ Breath and Respiration Analyzer (COBRA) inter- and intra-system reliability.

Measurement	Inter-System	Intra-System
VO_2_	0.825 [0.751, 0.881]	0.951 [0.927, 0.968]
VCO_2_	0.785 [0.684, 0.851]	0.876 [0.814, 0.912]
V_E_	0.857 [0.799, 0.899]	0.945 [0.920, 0.962]

**Table 4:** Percent coefficient of variation (%CV) for CO_2_/O_2_ Breath and Respiration Analyzer (COBRA).

Measurement	Total	Inter-System	Intra-System
VO_2_	7.2	8.5	4.3
VCO_2_	9.2	9.8	7.0
V_E_	6.6	7.6	4.7

## Data Availability

Data available upon reasonable request.
